# Relationship Between Reception of Low-Dose Computed Tomography Screening, Tobacco Cessation Attempt, and Reception of Pneumococcal Vaccine

**DOI:** 10.7759/cureus.23746

**Published:** 2022-04-01

**Authors:** Akesh Thomas, Zainab Fatima, Mohammad Darweesh, Debalina Das, Girendra Hoskere

**Affiliations:** 1 Internal Medicine, East Tennessee State University Quillen College of Medicine, Johnson City, USA; 2 Pulmonary and Critical Care Medicine, East Tennessee State University Quillen College of Medicine, Johnson City, USA

**Keywords:** low-dose ct screening, ldct, tobacco cessation, pneumococcal vaccine, lung cancer prevention

## Abstract

The stage at diagnosis is the single most important predictor of lung cancer outcome. Therefore, detecting lung cancer early is of utmost importance. Low-dose computed tomography (LDCT) has proven beneficial in the early detection and mortality reduction of lung cancer. Despite this, very few of the high-risk population get annual LDCT done. Patients' attitudes towards tobacco usage and preventive care can be a factor in getting LDCT. We analyzed the relationship between the willingness to undergo LDCT and a person's readiness to try tobacco cessation medication or get the pneumococcal vaccine. We also analyzed the relationship between patients who had tobacco cessation counseling and their willingness to get LDCT and pneumococcal vaccine. Medical records of high-risk patients seen in the East Tennessee State University (ETSU) clinics between January 1, 2016, and November 30, 2020, were analyzed retrospectively. In the data obtained, a total of 2,834 patients were current smokers and were included in the research. The study subjects were assessed in two ways, which from here on will be referred to as method one and method two. In the first method, patients who underwent LDCT were assessed, and the outcome investigated was tobacco cessation counseling, tobacco cessation medication prescription, and pneumococcal vaccination. In the second method, patients who had tobacco cessation counseling were assessed, and the outcome evaluated was LDCT, tobacco cessation medication prescription, and pneumococcal vaccination.

In the first method, out of 2,834 total population, 570 had undergone at least one LDCT screening during the study period. Of the 570 patients who underwent LDCT, 22.8% tried one of the tobacco cessation medications at least once during the study period (vs. 9.8% in patients who did not get the LDCT). Also, 71.5% of patients who had LDCT received at least one dose of pneumonia vaccine (vs. 35.5% in patients who did not get the LDCT). In the second method, 1,673 out of 2,834 patients received at least one tobacco cessation counseling, and out of those, 27.5% had LDCT screening (vs. 9.5% among those who never received counseling). Also, 54.9% received a pneumococcal vaccine (vs. 45.1% among those who did not receive counseling). The study demonstrates a relationship between getting LDCT and getting a pneumococcal vaccine or tobacco cessation medications. It also reveals that tobacco cessation counseling increases the odds of getting LDCT, tobacco cessation medications, and pneumococcal vaccine.

## Introduction

In the United States, lung cancer is the third most prevalent type of cancer and the leading cause of cancer-related mortality. About 135,720 lives were claimed by lung and bronchus cancers in 2020 in the US [1]. The number one prognostic factor for lung cancer is the stage at the time of diagnosis. The 5-year survival rate for non-small cell lung cancer is 63% for localized tumors and 7% for tumors with distant metastasis. Similarly, the 5-year survival rate for small cell carcinoma is 23% for localized tumors and 3% for tumors with distant metastasis [2]. This emphasizes the importance of early diagnosis and treatment of lung cancer.

As screening methods, chest radiography, low-dose computed tomography (LDCT), and sputum cytology have been studied. Among these techniques, the LDCT was the only effective method proven to lower the mortality rate. In 2011, the National Lung Screening Trial (NLST) demonstrated a 20% reduction in lung cancer-related mortality and a 7% reduction in all-cause mortality in high-risk patients after 6.5 years of follow-up with annual LDCT [3]. In 2019, the Nederlands-Leuvens Longkanker Screenings Onderzoek (NELSON) study demonstrated a 26% reduction in lung cancer-related mortality in men after 10 years of follow-up with volume-based LDCT [4]. In the US, the United States Preventive Service Task Force (USPSTF) recommends LDCT for adults between the ages of 50 and 80 who have smoked for at least 20 years and are either current smokers or former smokers who have quit within the last 15 years. As per this criterion, nearly 9 million Americans are eligible for lung cancer screening [5].

Despite all these proven benefits, only 5-10% of the eligible population tends to undergo LDCT [6]. The primary reason cited for this low rate of screening is the lack of insurance. Other factors, including the patient's lack of awareness, hesitancy to stop smoking, personal beliefs, and lack of adequate counseling and education from the physician, may also have contributed to the low screening rate. In this study, we analyze the data from a single center on the relationship between patients' willingness to undergo LDCT with three other variables: reception of pneumonia vaccine, tobacco cessation medication prescription, and tobacco cessation counseling. The paper also analyzes the relationship of receiving tobacco cessation counseling with the reception of LDCT, tobacco cessation medication prescription, and pneumococcal vaccination. This article was previously presented as an e-poster at the 2021 CHEST Annual Meeting held October 17-20, 2021.

## Materials and methods

Approval for the study was obtained beforehand from the East Tennessee State University (ETSU) institutional review board. Records of high-risk patients seen in the ETSU primary care clinics between January 1, 2016, and November 30, 2020, were analyzed retrospectively. Patients who were active smokers with more than 20 packs a year and between 55 and 80 years were included in the study (the age group was chosen as per the USPSTF recommendation during the study period). Patients with unknown duration of smoking and those with known lung cancer were excluded. Previous smokers who couldn't remember when they quit smoking were also excluded, although some of them might have met the screening criteria according to the USPSTF recommendations. The tobacco cessation medications included in the study were nicotine replacement therapy, varenicline, and bupropion, which were routinely offered to all patients who expressed interest in smoking cessation. Both pneumococcal conjugate vaccine-13 and pneumococcal polysaccharide vaccine-23 were counted as pneumococcal vaccines. The data were analyzed using SPSS Statistics, version 26.0 (IBM Corp., Armonk, NY) and Microsoft Excel (Microsoft Corp., Redmond, WA). A chi-square test was applied to calculate the statistical significance. The results are reported with a 95% confidence interval in brackets, with statistical significance defined as a p-value <0.001 unless otherwise specified. 

**Figure 1:**
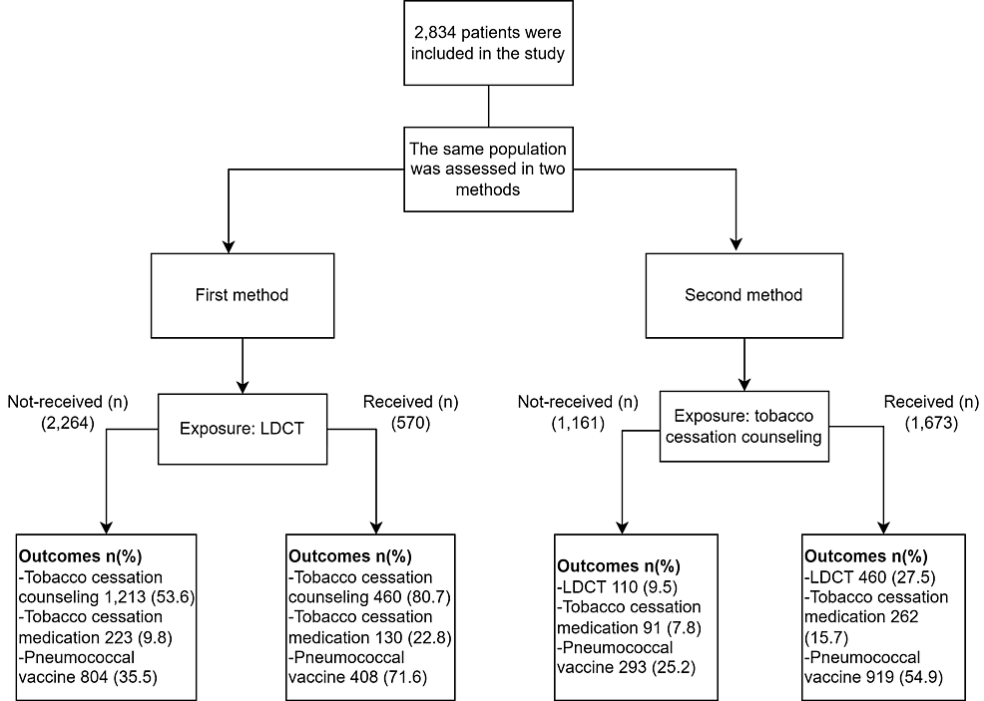
Study methodology. LDCT, low-dose computed tomography.

Patients who met the eligibility requirements were assessed in two ways. In the first method, patients who underwent LDCT were assessed, and the outcome investigated was tobacco cessation counseling, tobacco cessation medication prescription, and pneumococcal vaccination. In the second method, patients who had tobacco cessation counseling were assessed, and the outcome evaluated was LDCT, tobacco cessation medication prescription, and pneumococcal vaccination, as exhibited in (Figure 1).

## Results

Records of 11,136 patients aged between 55 and 80 years were studied, of which a total of 2,834 patients were current smokers with 20 or more packs a year. The median age of the study cohort was 63 (58-68). Characteristics of the study population and the overall rate of variables are given in Table 1. A total of 570 patients had gone through at least one LDCT screening during the study period (vs. 2,264 who did not have LDCT). Of the 570, 130 (22.8%) patients tried one of the tobacco cessation medications at least once during the study period (vs. 223 of 2,264 (9.8%) patients who did not get LDCT), with an odds ratio (OR) of 2.70 (95% CI: 2.12-3.4). Furthermore, 408 of the 570 (71.5%) patients received at least one dose of pneumococcal vaccine (vs. 804 of 2,264 (35.5%) patients who did not have LDCT), OR of 4.57 (95% CI: 3.73-5.59). Also, 460 of the 570 (80.7%) patients had received tobacco cessation counseling (vs. 1,213 of 2,264 (53.5%) patients who did not get LDCT) (Table 2).

**Table 1:** Characteristics of the study population.

Exposure	Received n(%)	Not received n(%)
CT chest screening history	570 (20.1)	2264 (79.9)
Pneumonia vaccine	1212 (42.8)	1622 (57.2)
Tobacco cessation counseling	1673 (59)	1161 (41)
Tobacco cessation medication	353 (12.5)	2481 (87.5)

**Table 2:** Association of various factors with LDCT. *Statistically significant

Variable	CT chest screening history		Χ^2^	P-value	OR (95% CI)
	Yes (n=570)	No (n=2264)			
Pneumonia vaccine, n(%)					
Received	408 (71.6)	804 (35.5)	241.99	<0.001*	4.57 (3.73-5.59)
Not received	162 (28.4)	1460 (64.5)			
Tobacco cessation counseling, n(%)					
Received	460 (80.7)	1213 (53.6)	138.52	<0.001*	3.62 (2.89-4.53)
Not received	110 (19.3)	1051 (46.4)			
Tobacco cessation medication, n(%)					
Received	130 (22.8)	223 (9.8)	70.10	<0.001*	2.70 (2.12-3.43)
Not received	440 (77.2)	2041 (90.2)			

In the second analysis, which studied the effect of tobacco counseling on LDCT, tobacco cessation medication prescription, and pneumococcal vaccination, 460 of 1,673 (27.5%) patients who got tobacco cessation counseling underwent LDCT, compared to the 1,161 patients who never got tobacco counseling; only 110 of them (9.5%) underwent LDCT (OR: 3.62, 95% CI: 2.89-4.53). Similarly, 262 of 1,673 (15.6%) patients who got tobacco cessation counseling received tobacco cessation medication (vs. 91 of 1,161 (7.8%) among those who never underwent any counseling). In addition, 919 of 1,673 (54.9%) of those who got tobacco cessation counseling received a pneumococcal vaccine (vs. 293 of 1,161 (25.2%) among those who never underwent any counseling) (Table 3).

**Table 3:** Association of various factors with tobacco cessation counseling. *Statistically significant

Variable	Tobacco cessation counseling		Χ^2^	P-value	OR (95% CI)
	Yes (n=1673)	No (n=1161)			
CT chest screening history, n(%)					
Yes	460 (27.5)	110 (9.5)	138.52	<0.001*	3.62 (2.89-4.53)
No	1213 (72.5)	1051 (90.5)			
Pneumonia vaccine, n(%)					
Received	919 (54.9)	293 (25.2)	246.90	<0.001*	3.61 (3.06-4.25)
Not received	754 (45.1)	868 (74.8)			
Tobacco cessation medication, n(%)					
Received	262 (15.7)	91 (7.8)	38.46	<0.001*	2.18 (1.69-2.80)
Not received	1411 (84.3)	1070 (92.2)			

## Discussion

This study demonstrates a clear positive correlation between receiving LDCT with tobacco cessation medication and pneumococcal vaccine reception. It was also possible to demonstrate that receiving tobacco cessation counseling is independently related to receiving both LDCT screening and tobacco cessation medication. It is obvious that patients who are hesitant to quit tobacco smoking will generally not be receiving tobacco cessation medications, which manifests those patients' unwillingness. Likewise, patients with inadequate health literacy and poor health education will not be receiving pneumonia vaccines or tobacco cessation medications. Lack of tobacco cessation counseling at each visit is an indicator of inadequate health education.

The combination of lung cancer screening with smoking cessation counseling yielded a 9% reduction in cumulative lung cancer mortality in a US region with a relatively high smoking prevalence and lung cancer incidence [7]. Furthermore, people with abnormal LDCT findings had even higher cessation rates because they were more receptive to making lifestyle changes [8]. The fact that a higher proportion of people who received tobacco cessation counseling chose tobacco cessation medication emphasizes consistent and efficient guidance and good patient communication. But smokers are more likely to contract community-acquired pneumonia and invasive pneumococcal disease; therefore, it is crucial to educate them about the importance of timely vaccination [9]. According to some previous studies, healthcare provider recommendations can boost vaccination rates by up to 80% among patients who had previously declined vaccination [10]. Low socioeconomic status has already been described as associated with increased tobacco smoking [11], leaving this population at particular risk of poor health literacy and a low rate of LDCT reception. The 2021 change in USPSTF guidelines extended the age window from 55-80 years to 50-80 years, so persons aged 50-55 also became eligible for LDCT, further increasing the number of people in the community eligible for LDCT.

It should, however, be noted that the single-center retrospective nature limits the study. The study population of East Tennessee has a higher smoking rate and low average education compared to the national indices, even though the results can grossly be similar across the whole country. Yet another limitation of the study is that tobacco cessation counseling may not always be documented by the physician, even if it is done. The quality of the tobacco cessation counseling is also a nonmeasurable entity with the study's retrospective nature.

## Conclusions

This study concludes that there is an association between LDCT screening and the patient's willingness for tobacco cessation and pneumococcal vaccination. Similarly, reception of tobacco cessation counseling is independently associated with receiving LDCT, tobacco cessation medications, and pneumococcal vaccination. A more detailed, prospective analysis is needed to better demonstrate the predictors for the reception of LDCT to modify and implement healthcare delivery policies effectively.
